# Behavior Modulates Effective Connectivity between Cortex and Striatum

**DOI:** 10.1371/journal.pone.0089443

**Published:** 2014-03-11

**Authors:** Alexander Nakhnikian, George V. Rebec, Leslie M. Grasse, Lucas L. Dwiel, Masanori Shimono, John M. Beggs

**Affiliations:** 1 Program in Neuroscience, Indiana University, Bloomington, Indiana, United States of America; 2 Department of Psychological and Brain Sciences, Indiana University, Bloomington, Indiana, United States of America; 3 Cognitive Science Program, Indiana University, Bloomington, Indiana, United States of America; 4 Department of Physics, Indiana University, Bloomington, Indiana, United States of America; 5 Department of Physical and Health Education, Graduate School of Education, The University of Tokyo, Tokyo, Japan; Centre national de la recherche scientifique, France

## Abstract

It has been notoriously difficult to understand interactions in the basal ganglia because of multiple recurrent loops. Another complication is that activity there is strongly dependent on behavior, suggesting that directional interactions, or effective connections, can dynamically change. A simplifying approach would be to examine just the direct, monosynaptic projections from cortex to striatum and contrast this with the polysynaptic feedback connections from striatum to cortex. Previous work by others on effective connectivity in this pathway indicated that activity in cortex could be used to predict activity in striatum, but that striatal activity could not predict cortical activity. However, this work was conducted in anesthetized or seizing animals, making it impossible to know how free behavior might influence effective connectivity. To address this issue, we applied Granger causality to local field potential signals from cortex and striatum in freely behaving rats. Consistent with previous results, we found that effective connectivity was largely unidirectional, from cortex to striatum, during anesthetized and resting states. Interestingly, we found that effective connectivity became bidirectional during free behaviors. These results are the first to our knowledge to show that striatal influence on cortex can be as strong as cortical influence on striatum. In addition, these findings highlight how behavioral states can affect basal ganglia interactions. Finally, we suggest that this approach may be useful for studies of Parkinson's or Huntington's diseases, in which effective connectivity may change during movement.

## Introduction

It is tremendously important to understand interactions among the subcortical nuclei of the basal ganglia (BG) because these nuclei have been implicated in a broad array of motor and higher-order cognitive functions like reward seeking [1]–[3], habit formation [4], action selection [5], and multi-modal information integration [6], [7]. In addition, dysfunction in the BG and related systems is thought to play a causative role in Huntington's and Parkinson's diseases [8]–[13].

However, it has been extremely challenging to understand interactions in the BG because these structures have many recurrent synaptic connections [7], [13]. Numerous reports also indicate that activity in the BG nuclei is strongly modulated by behavioral states [14]–[16], suggesting that interactions within the BG may change rapidly with behavior.

An attractive solution to this dilemma would be to study just one BG connection, and to observe how interactions in that connection change with different behaviors. In this respect, perhaps the simplest connection in the BG is that between the cortex and the striatum. Neocortex sends direct, monosynaptic projections to striatum [13], the input nucleus of the BG. Moreover, striatum does not project directly back to cortex. Striatum can influence cortex indirectly, though, by way of projections from striatum to other BG nuclei, which in turn project to thalamus, which then projects to cortex [7]

To date, studies of interactions between cortex and striatum have focused on correlated activity using both spike trains [17] and local field potentials (LFPs) [18]. While studies of correlated activity are highly informative, determining the contributions of cortical and subcortical processes to such correlations requires methods to distinguish directed influence from temporal synchrony. To our knowledge, only two studies have assessed directed relationships in the cortico-striatal pathway of rats. Other groups [19] used directed measures to explore interactions in anesthetized rats and found that cortical influence over BG nuclei exceeded reciprocal influence. In another study, the investigators used directed measures during spike and wave seizures in a rat model of epilepsy, and again found cortico-striatal influence to be dominant [20]. Building on this important work, we sought to measure how different free behaviors might modulate directional influence between cortex and striatum in healthy animals.

To do this, we characterized directed influence between cortex and striatum using LFPs recorded in primary motor cortex (M1) and dorsal striatum (dStr) from awake, unrestrained rats. In addition to observing free behaviors, we recorded during sleep and anesthesia in order to compare rest states to spontaneous behavior and to each other, and to assess the neural correlates of drug-induced and natural inactivity. To gauge directional network interactions, we applied Granger causality (GC) [21]–[23], which quantifies the degree to which two systems exchange directed information based on temporal precedence. GC is a well-established effective connectivity metric and has shown great promise based on foundational work in both animals [24] and humans [25]. The mathematical underpinnings of GC are described in detail elsewhere [22], [26], [27]. Importantly, GC is easily expanded into Fourier space, making it an ideal method for studying LFPs, which are rich in spectral content. Recent findings suggest that this method is well-suited to analysis of electrophysiological signals [20].

Given that M1 has massive glutamatergic projections directly to dStr, we expected that GC would be very strong in the corticostriatal direction in all phases of behavior. Because dStr is connected to M1 only indirectly, by way of several basal ganglia nuclei and then the thalamus, we expected GC would be relatively weak in the striatocortical direction. Consistent with previous results [19], [20], we found that cortical influence exceeded striatocortical drive during natural inactivity and anesthesia. During voluntary movement, however, striatal influence over cortex increased markedly and became as strong as cortical influence over striatum in certain frequency bands. To our knowledge, this is the first report that striatal influence over cortex can be as strong as cortical influence over striatum. This work highlights the bidirectional nature of influence between cortex and striatum, and emphasizes the importance of recording from naturally behaving animals for understanding basal ganglia function.

## Methods

All procedures adhered to NIH guidelines regarding use of animals in research, and were approved by the Institutional Animal Care and Use Committee (Assurance Number A4094-01).

### Electrodes

Electrodes were constructed in-house by friction fitting gold socket connectors to 75 micron stainless steel, Formvar insulated microwires. Electrode assemblies consisted of eight microwires, four each to be implanted in M1 and dStr. As M1 lies just dorsal to dStr, we were able to target both structures simultaneously by combining all the microwires into a single bundle and cutting the cortical wires 4 mm shorter than the striatal wires. Relative distances between electrodes were determined from a stereotaxic atlas [28] and precise wire length was accomplished by cutting the wires under a microscope using a sharp pair of surgical scissors. Though we only analyzed a single trace for each structure, we implanted multiple electrodes to minimize data loss due to individual electrode failure or heavy noise on a particular channel. Channels were inspected for artifacts and the one to be analyzed in each structure was selected before data were analyzed. Power, coherence, and causality spectra were not known when selecting data for analysis.

### Surgery and Electrophysiology

Animals were anesthetized with a mixture of ketamine (80 mg/kg) and xylazine(10 mg/kg). A single hole was drilled over both M1 (−0.5 mm) and dorsal striatum (−4.5 mm) (bregma, +1.6 AP, +/−2.5 ML). The dura was plucked with a sharp, angled needle with great care not to disrupt the underlying cortex. Three to four additional holes were drilled into but not through the skulls. Screws inserted into these holes were anchored to the electrode using dental acrylic. All LFPs were referenced to the potential of a screw near the midline and contralateral to the recording electrodes, following prior studies [29]; this screw also served as the animal ground.

Recording sessions occurred between 9:00 AM and 5:00 PM during the light cycle. Animals were placed in an open field consisting of a 48 cm L×26 cm W×20 cm H Plexiglas cage with a cardboard floor in lieu of standard bedding to minimize static noise. The electrode assembly was connected to a flexible harness equipped with field-effect transistors that provided unity gain current amplification across all eight recording electrodes. The open field was placed in an electrically grounded sound-attenuating Faraday cage. Extracellular electrical activity was routed through multi-channel preamplifiers that provided 500X gain and 0.9 to 300 Hz bandpass filtering (Plexon, Inc. Dallas, TX). Signals were digitized at 1 kHz and recorded (Multichannel Acquisition Processor, Plexon). Data were stored on an external hard drive and transferred to a Hewlett-Packard desktop computer for analysis. For each recording session, the trace with the fewest artifacts based on visual inspection was selected from each structure. Traces to be analyzed were selected before any analysis was performed. These data were transferred to Matlab (The Mathworks, Natick, MA) for analysis. Animals were awake and unrestrained during the first thirty to forty-five minutes of the recording session. The same animals were then administered a ketamine/xylazine mixture at half a surgical dose and placed on Deltaphase isothermal pads to provide supplemental heat. Recordings continued until the animal exhibited spontaneous movement indicating incomplete anesthesia. Anesthesia lasted 10–30 min, depending upon individual variations in drug sensitivity. For consistency, only the first 10 min of anesthesia from each animal were analyzed. We discarded data gathered after the first sign of incomplete anesthesia, such as a response to a paw pinch or a whisker flick.

### Histology

**Figure 1:**
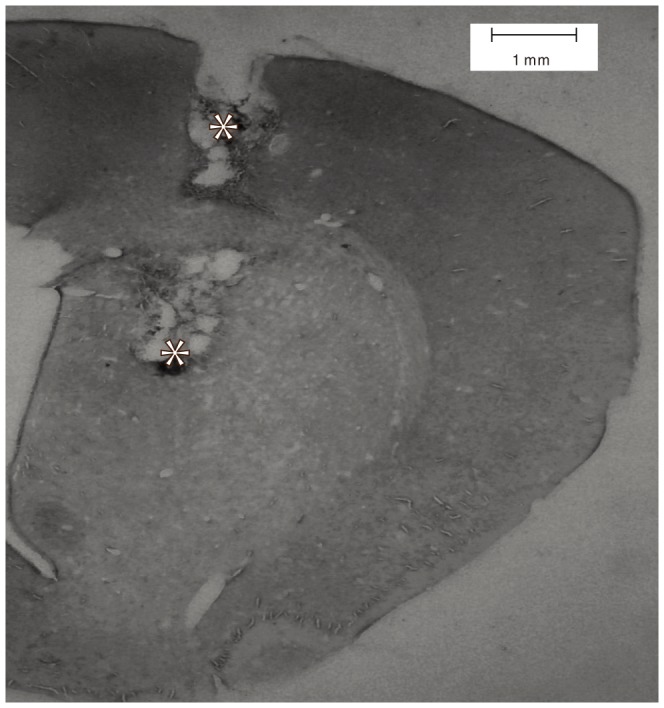
A representative histology section. Asterisks indicate the location of electrode tips, as revealed by metal ion deposits marked by ferrocyanide.

Histology was performed as previously described [11], [30]. Briefly following recording, animals were deeply anesthetized using a ketamine/xylazine mixture at double a surgical dose. Electrolytic lesions were produced at the electrode implantation sites by applying a 50 microamp current across a randomly selected working electrode and all three of its neighbors for three seconds each. Animals were transcardially perfused with saline followed by a 10% solution of potassium ferrocyanide in neutral, buffered formalin. Brains were extracted and cryoprotected in 30% sucrose dissolved in formalin for at least 24 hours. 80-micron coronal sections were obtained on a microtome and inspected to verify correct electrode implantation (Fig. 1).

### Behavioral Analysis

Data sets were separated according to behavioral epochs. Occurrences of different behaviors were identified offline by two research assistants trained to recognize each behavior of interest. Coding consistency was determined by comparing the coded behaviors observed by each coder to data coded by the primary author. Near perfect (∼95%) agreement between these data sets was obtained before coders produced data for analysis. For all coded data sets, the primary author inspected a random sample of the coders' time stamps to ensure continued reliability across the experiment. Coders were instructed to use conservative standards and indicate when a particular behavioral epoch was ambiguous. In these cases, the decision to include a trial in the final analysis was made by the primary author prior to analysis of the data sets.

We analyzed the following spontaneous behavioral events: rearing (when the rat's front two paws were off the bottom of the cage), immobile alertness (when the rat was sternal and passively observing its surroundings with no gross motor output), generalized exploration (when the rat was actively moving about the cage but stopping every two to three steps to observe its surroundings), and sleep (characterized by a curled posture and complete lack of movement lasting at least one minute). Data gathered during free behaviors – excluding sleep – were divided into 2 sec epochs and pooled into ensemble averages of at least ∼90 and at most ∼200 events. If a behavioral epoch lasted longer than 2 sec, only the first two seconds were used for analysis in order to maintain a consistent spectral resolution across all trials and behaviors. We chose 2 sec epochs so as to ensure that only one behavior was expressed during each interval to be analyzed. This conservative approach to categorizing behavior did not compromise the analyses we could perform on these 2 sec epochs, as explained below. Data recorded during sleep and anesthesia was treated as long stationary trials. We recognize that sleep and anesthesia are not homogenous states and that it is possible for the neuronal signal to exhibit different spectral properties at different phases of anesthesia and sleep. We confirmed that the spectral decomposition was consistent across trials using a multiwavelet method [31], [32]. Briefly, this method computes unbiased single-trial power and coherence using a weighted average of mutually orthogonal eigenspectra. Using this method, we are able to construct spectrograms without the use of a sliding window or multitrial average. We confirmed that the spectral content of the LFPs did not vary across sleep and anesthesia (see results for representative spectrograms). These data sets were divided into five-second periods separated by two-second buffers to reduce serial correlations. As very low-frequency oscillations (1–4 Hz) are known to characterize in-vivo LFPs recorded during anesthesia and sleep, we chose a longer window in order to better resolve very low frequency components. This was feasible for sleep and anesthesia since these data sets can be arbitrarily divided into segments of any length. Data gathered during spontaneous behavior, conversely, are constrained by the average amount of time the rats spent engaged in a particular behavior.

### Signal Processing and LFP Analysis

Data sets were downsampled to 240 Hz using an in-house linear interpolation algorithm. Power spectral densities were inspected both before and after downsampling to guard against aliasing. Power at the native sampling rate vanished before the downsampled Nyquist frequency of 120 Hz, obviating the need for an anti-aliasing filter. Power line noise at 60 Hz was reduced using a multitaper method [33]; this method is most effective when the sampling rate is an integer multiple of the noise frequency, hence our chosen downsampling method. Epochs contaminated with obvious artifacts or for which the behavioral state was ambiguous were removed prior to any analysis. Coders were blind to the behavioral state corresponding to a particular epoch while removing artifacts in order to guard against biased data selection. Epochs were detrended, the ensemble mean was subtracted point wise, and each data point was divided by the temporal standard deviation to give all trials equal weights in the ensemble averages. Our use of LFP signals follows that of previous investigators in the basal ganglia [14], [34] and in cortex [35]–[37], who have reported that LFPs can be used to indicate regional brain activation at a scale that is intermediate between single units and fMRI.

### Spectral Estimation and Granger Causality

We selected GC from among multiple available causality metrics, as it is well characterized and suitable for our experimental design. Unlike dynamic causal modeling [38], GC does not require a prior model, making it well-suited to exploratory experiments. Although Schreiber's transfer entropy [39] admits a wavelet-based representation in scale space that maps directly to frequency [40], applying transfer entropy to continuous signals raises a number of technical issues [41], [42]. GC, in contrast, derives directly from standard linear systems theory [27] and naturally describes continuous systems.

The mathematical details of GC are well described in Ding et al., 2006 and Seth, 2010. In simple terms, GC attempts to quantify how much knowledge of system A's past improves the ability to predict system B's future. Usually, knowledge of B's past can provide some ability to predict B's future. For example, knowing that the amplitude of local field potential B has been increasing for the past 10 milliseconds usually will provide some information about how B's amplitude will behave in the next 10 milliseconds. If knowledge of system A's past further improves this prediction (beyond some small amount that would be expected by chance), then we say that A is “Granger causal” to B. To continue the example, when an increase in amplitude of LFP A often precedes an increase in amplitude of LFP B, then we say that A is Granger causal to B. Granger causality is used quite often [43] in neuroscience studies [24], [25], [44] as a way of assessing influence, regardless of patterns of synaptic connectivity.

Power, coherence, and GC were obtained by fitting an autoregressive model to each trial and averaging over all model coefficients. This is a standard procedure for estimating ensemble properties such as power, and it was implemented with the BSMART toolbox [43]. We pooled all the data for each behavior into the same ensemble and combined data from all five animals into the spectral estimate for each behavior. We performed an inter-subject variability analysis to validate this procedure (see below). The covariance matrix is similarly constructed by estimating the temporal variance of the model residuals and averaging these values across trials. Power and coherence were derived from the regression coefficients using standard parametric spectral estimation, and GC was derived using methods described by Ding and colleagues [27]. We determined the model order, which is the number of lagged observations used to construct the regression model, by minimizing the Bayesian information criterion (BIC) as a function of parameter number. Brovelli et al. (2004) report that the commonly used Akaike Information Criterion failed to converge in their study of cortical LFPs in the monkey, and Ding et al. (2006) suggest the use of either the AIC or BIC depending upon the particulars of the data. With this in mind, we chose the more conservative BIC to determine the model order. The BIC is minimized at an optimal trade-off between model accuracy and over-fitting [45]. The BIC generally converged at high model orders (from 40 to 49 model parameters, Table 1). As overparamaterization can introduce spurious spectral peaks [46], we analyzed the data sets using multiple model orders (data not shown) to ensure robustness against variation in the number of regression parameters. Furthermore, we compared parametric and non-parametric GC spectra (see results) in order to ensure that idiosyncrasies of model fitting did not substantially influence our analysis. To estimate non-parametric GC, we used code provided by Mukeshwar Dhamala to implement the derivation of GC directly from the spectral matrix. Power and coherence were estimated using standard Fourier analysis. For each trial, we obtained the energy of individual signals (the squared modulus of the Fourier transform) and cross-spectral density (the product of the Fourier transform of X times the complex conjugate of the Fourier transform of Y, and vice-versa). The resulting spectral estimates were averaged across all trials of a particular behavior in order to obtain the proper ensemble average. The resulting matrix was factored using a recursive algorithm to obtain the transfer matrix and residual covariance matrix. Once these matrices are defined, GC is estimated exactly as it is in the parametric case. If poor model order selection compromised our results, we would expect the parametric and nonparametric analyses to differ substantially, which they do not.

**Table 1:** Peak Granger causality for each behavior (shown in bold) with the 95% intersubject confidence interval, determined by a bootstrapping procedure described in the methods.

Behavior	Number of Trials	Model Order	Peak GC with 95% CI
Anesthesia	252 (5 Sec)	40	M1->dStr
			3 Hz [0.320, **0.33**, 0.339]
			8 Hz [0.057, **0.063**,0.067]
			15 Hz [0.015, **0.018**, 0.021]
			dStr->M1
			3 Hz [0.071, **0.077**, 0.082]
			8 Hz [0.048, **0.053**, 0.057]
			15 Hz [0.028, **0.031**, 0.035]
			
Sleep	301 (5 Sec)	48	M1->dStr
			4 Hz [0.157, **0.171**, 0.178]
			dStr->M1
			No Peaks
			
Alert and Inactive	229 (2 Sec)	49	M1->dStr
			2 Hz [0.087, **0.11**, 0.178]
			8 Hz [0.036, **0.044**, 0.054]
			dStr->M1
			6 Hz [0.006, **0.009**, 0.014]
			
Exploration	103 (2 Sec)	40	M1->dStr
			2 Hz [0.095, **0.126**, 0.172]
			8 Hz [0.046, **0.058**, 0.077]
			dStr->M1
			9 Hz [0.043, **0.06**, 0.079]
			21 Hz [0.035, **0.044**, 0.057]
			35 Hz [0.015, **0.024**, 0.028]
			
Rearing	93 (2 Sec)	40	M1->dStr
			2 Hz [0.056, **0.084**, 0.11]
			8 Hz [0.011, **0.018**, 0.028]
			dStr->M1
			9 Hz [0.047, **0.071**, 0.094]

The second column shows the number of realizations used to generate the ensemble average, and the duration of each realization in parentheses. Model order refers to the number of parameters used to generate the regression model, using data from all animals engaged in a particular behavior.

#### Parametric GC

Granger's original work followed directly from early insights by Weiner that control of one system over another should manifest as increased predictive power when using lagged observations of one system – a putative cause – to predict the future states of another. Calling one system X and the other Y, we conclude that X “causes” Y, in Granger's sense, if observing the past of X improves our ability to predict the future of Y *after* we have already considered the past of Y. Granger codified this intuitive concept of causality using autoregressive modeling of time series. The following system of equations facilitates a mathematical quantification of the predictive power of both X and Y over one another:
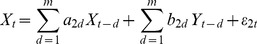
(1)

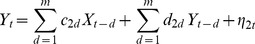
(2)In Eqs. 1 and 2, upper case letters are values of each time series, lower case letters are regression coefficients, m is the model order (estimated by minimizing the Bayeisan or Akaike information criterion), and Greek letters are the temporal model residuals. Comparing the variance of the residuals from the above equations to those obtained in a univariate model (one that factors the past of one time series alone) allows us to derive temporal GC. Here, we provide only the derivation of spectral GC, as it is the focus of this paper. We begin by rearranging Eqs. 1 and 2 such that only the residuals remain on the RHS.
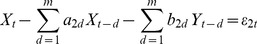
(3)

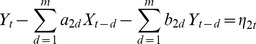
(4)Fourier transforming both sides of the above equations and recasting them as a vector-valued system yields:
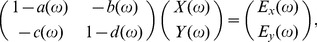
(5)where, 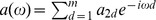, and the other spectral regression coefficients are defined in the same manner. The spectral GC from X to Y is, intuitively, the portion of X's power at frequency ω accounted for by Y. To obtain this decomposition, we express the Fourier transforms of both time series in terms of the spectral model residuals. Multiplying both sides of Eq. 5 by the inverse of the coefficient matrix yields:
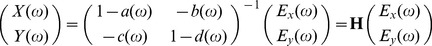
(6)
**H** is the transfer matrix, which maps the amplitude and phase of the residuals to the spectral representations of X and Y on the LHS of Eq. 6. In this formulation, we treat each residual term as a “drive” acting upon the oscillators X(ω) and Y(ω). To derive the spectral causality from X to Y, consider the definition of Y(ω) given by Eq. 6:
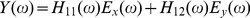
(7)It is clear from the above equation that Y(ω) admits a representation in terms of both model residuals, one arising from X and the other from Y. The final step is to represent the power (instead of the raw Fourier transform) of the signal in terms of X and Y. Note that multiplying both sides of Eq. 7 by its own complex conjugate transpose yields:
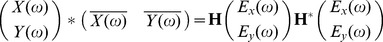
(8)For a single trial, the above definition gives the energy of X and Y in terms of the errors and when averaged over multiple realizations of the same event yields the spectral matrix (**S**). The diagonal entries in **S** are auto-spectra and off-diagonal entries are cross-spectra. After such averaging, we represent the spectral matrix as follows:
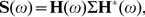
(9)where **Σ** is the covariance matrix of the model residuals. Following a normalization introduced by Geweke, which sets the residual covariance equal to zero and produces a modified transfer matrix, 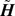, we can decompose the power spectrum of Y into exactly two terms, one accounting for Y's self-influence and one reflecting causal influence exerted by X. We obtain the following representation for the auto-spectrum of Y:
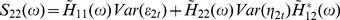
(10)which gives rise to the definition of GC from X to Y:
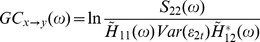
(11)It is illustrative to consider the case of zero causality. When X exerts no influence over Y, then the second term on the RHS of Eq. 10 vanishes and the power of Y at ω is exactly equal to the denominator in Eq. 11. In this case, GC = ln(1) = 0. As the causal power of X over Y increases, the argument of the logarithm becomes greater than one, and GC increases accordingly. For a full derivation of GC see Ding et al. (2006). Other derivations are provided elsewhere [24], [47].

#### Non-Parametric GC

Estimating GC without deriving a parametric model hinges on constructing Eq. 9 without utilizing autoregression. Dhamala and colleagues [48] provide an elegant solution to this problem based on Wilson's analysis of a class of matrices, to which **S**(ω) belongs, for which each member admit the following decomposition into a set of unique minimum phase functions [49]:
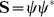
(12)ψ(z) is defined in Fourier series on S^1^ as:
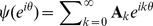
where 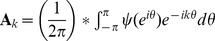. Wilson provides a recursive algorithm, implemented using code written by Dhamala and colleagues, which converges to the theoretical value of ψ. Importantly, ψ admits a holomorphic extension to the inner disk. This property enables us to define **H**, **S**, and ψ as functions taking any complex input {|z|≤1}. It can be shown that **H**(0) is the identity matrix, thus by Eq. 9 **S**(0) is the covariance matrix. Considering **H**(0) and **A**(0), a real upper triangular matrix with positive diagonal entries, we have the following definition of the covariance matrix.
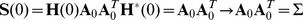
To obtain the transfer matrix, we rewrite Eq. 12 as:
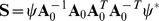
The terms between the outer most functions reduce to the identity matrix times itself, hence the above equation is exactly equal to Eq. 12. Compare the above expression for **S** with Eqs. 9 and 12. It is clear that the transfer matrix is defined as follows:
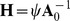
Having expressions for the covariance matrix and transfer matrix, we are able to proceed as in the parametric case and decompose the diagonal entries in **S** into intrinsic and causal terms. GC is defined as the logarithm of the ratio of a signal's power to its intrinsic (non-causal) power. Dhamala's formulation gives rise to an inherently spectral metric; however, temporal causality is bounded from above by the integral of spectral GC over a complete cycle:
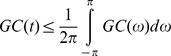
Under general conditions, the equality holds. Hence, temporal GC can be reconstructed, if desired, using this method.

### Significance Testing

Statistical significance for coherence and GC was estimated by independently permuting the time series for each data set 1000 times. For example, to estimate the statistical cutoffs for rearing, we randomly rearranged all 93 2 sec epochs such that the temporal precedence patterns were destroyed (i.e. we might estimate the causality from a cortical signal recorded during trial one to a striatal signal recorded during trial fifty). This procedure maintains the statistical properties of the data set, such as mean and variance, while randomizing the temporal relationships underlying causality estimation. We estimated cutoffs for anesthesia in sleep in a similar manner, the only difference being that we used 5 sec instead of 2 sec epochs, as these data sets were divided into 5 sec trials to maximize low frequency spectral resolution. When estimating the cutoff values, we used the same model order as was used in the analysis of non-permuted data (see Table 1). This procedure allows us to obtain the peak values of GC under the null hypothesis that the observed causality is due to chance. As GC is asymmetric, two cutoffs are needed to assess its significance and we used Bonferroni's correction for multiple comparisons. Surrogate data underwent the same signal processing procedures as non-randomized trials. For both coherence and GC, we generated vectors of 1000 elements containing the maximum coherence or GC value obtained for each randomized trial. Cutoff values were obtained by taking the 0.995 quantile of this vector, generating confidence limits with a 0.005 significance level. This is a well-established procedure for spectral GC, which does not have a known theoretical null distribution. Coherence cutoffs obtained in this manner were close to the theoretical value of the coherence between random Gaussian processes derived by Brillinger [50], 1−(1−p)^1/(M−1)^, where M is the number of data segments used in the ensemble estimate and p is the significance level.

We generated a coherence cutoff for each behavior by randomly dividing all the data into epochs lasting as long as the analysis window for a particular behavior and randomly pairing these surrogate trials. For instance, we generated the cutoff for coherence during sleep by randomly sampling 301 trials, each lasting five seconds, and analyzing them using a model order of 48. This procedure allowed us to estimate the null distribution obtained under the assumption that the distribution of coherence per frequency bin is independent of behavioral state. For GC, individual cutoff values were generated for each behavior by randomly matching different trials of the same behavior to generate a null distribution of data that has the same statistical and spectral properties as the original set, but for which the temporal concordance between time series was randomized.

### Inter-subject Variability

Spectral estimation from ensemble averages hinges on the assumption that each individual trial is a realization of the same underlying process. Depending upon the behavior, we observed at least 93 and at most 301 realizations of the same behavior. Given the number of available trials, it is likely that our data provides a reasonable estimate of ensemble properties, such as power and coherence. We should, however, consider the possibility that individual variations among animals biased the spectral estimation metrics. To control for this, we performed a bootstrap analysis in which the contribution of each individual animal was randomly amplified or attenuated. For each data set, we removed approximately one quarter of the trials. Trials to be removed were randomly selected using Matlab's built-in randperm function and the analysis was constructed such that data from any given animal was equally likely to be removed. Each surrogate data set was centered and standardized using the mean and standard deviation of the “pruned” data set. This analysis allowed us to determine upper and lower bounds on the distribution obtained when the individual contribution of any one animal is randomized. We repeated this procedure 1000 times. As power, coherence, and GC are average values, the central limit theorem should apply in theory assuring normality of resampled distributions. We found, however, that for different data sets anywhere from 10%–25% of resampled sets failed the Lilliefors test of normality [51]. Therefore, in lieu of using the mean and standard deviation, we obtained cutoff values by adding and subtracting the 97.5^th^ and 2.5^th^ percent empirical quantile from the mean of each surrogate distribution. The results of this analysis are summarized in Table 1.

### Trial-to-Trial Variability Analysis

As power, coherence, and spectral GC are inherently ensemble properties, naively estimating variance as the expected difference between data points and their mean is not appropriate. Consider power, which is the average squared amplitude of each Fourier coefficient. Taking the mean squared difference between squared amplitudes and their average gives the expected difference between energy and power, not the true variance of the power spectrum. To circumvent this issue, we once again applied a bootstrapping procedure, resampling with replacement 1000 times from all the trials for a particular behavior. For each data set, we randomly sampled N trials, where N is the number of events obtained for each behavior type. Power, coherence, and GC were then calculated by taking the appropriate ensemble averages over the resampled data sets. This procedure approximates drawing multiple samples from the same underlying population and allows us to set confidence limits when the individual data points are themselves average values.

## Results

### General Observations

**Figure 2:**
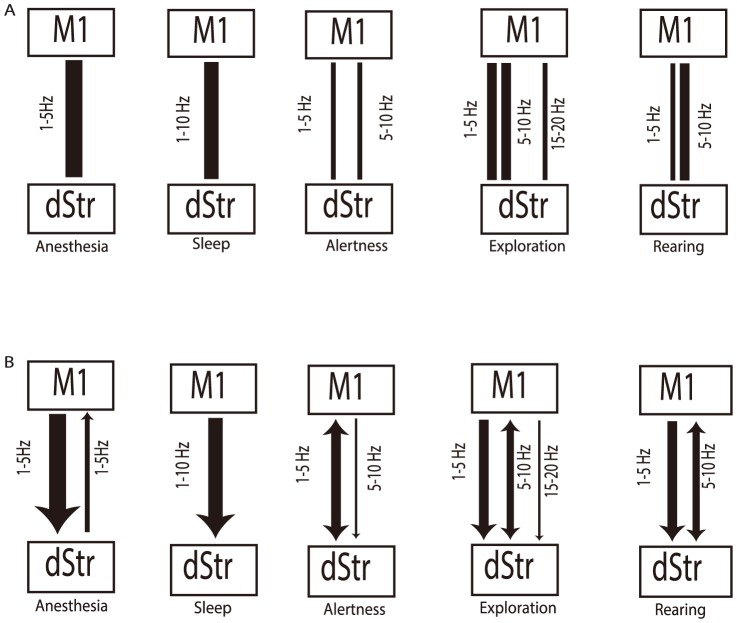
A schematic representation of coherent interactions and causal dynamics revealed by Granger causality. Corticostriatal interactions during multiple behavioral states as revealed by coherence analysis (A) and Granger causality (B). Line thickness is proportional to the peak coherence or causality value and inset labels indicate the corresponding frequency range. For legibility some weak connections are omitted in this plot; see Figs. 4 and 5 for full spectral decompositions. Arrowheads in B indicate direction of influence; these are omitted in A, as coherence values are symmetric with respect to direction. Note the substantial refinements afforded by Granger causality, which reveals both symmetric and asymmetric driver/receiver dynamics giving rise to the coherence spectra as well as the contributions of corticostriatal and striatocortical pathways to different peaks in the coherence spectra.

**Figure 3:**
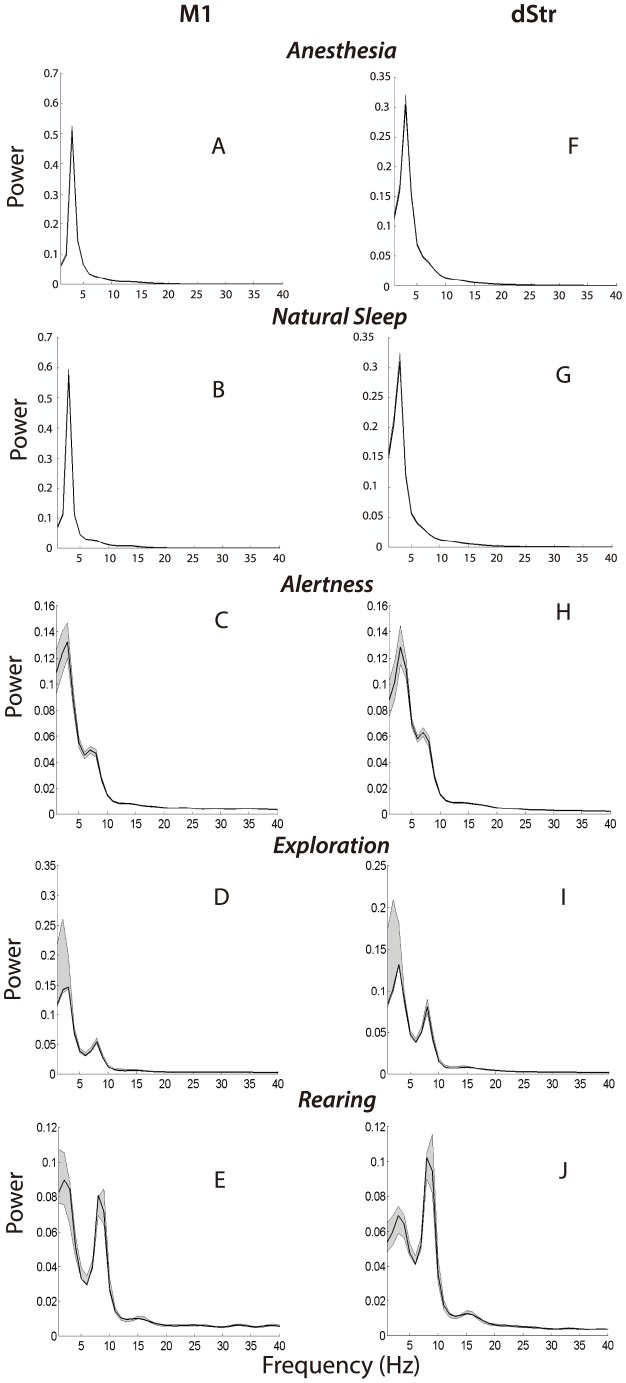
f Power spectral densities generated using LFPs recorded in M1 (A–E) and dStr (F–J) during anesthesia (row 1), sleep (row 2), immobile alertness (row 3), exploration (row 4), and rearing (row 5). Power in both M1 (A) and dStr (F) was higher during anesthesia than during sleep (B,D). Quantile ranges for some data are too narrow to be discernable in this figure. Gray shaded regions indicate a non-Gaussian approximation of +/−1 standard deviation based on resampling (see methods). Note the variation in contributions to total power from oscillations in the 1–5 Hz and 7–12 Hz ranges across the three spontaneous behaviors.

**Figure 4:**
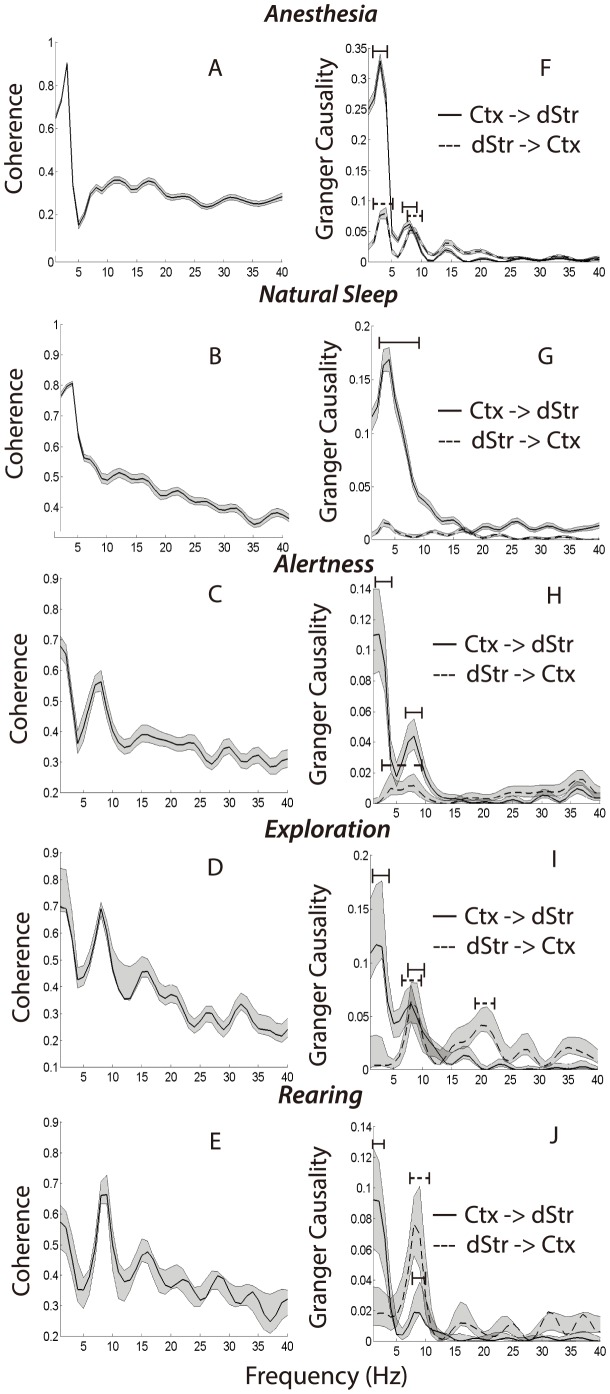
Coherence and causality spectra during multiple behaviors. Coherence spectra (A–E) and Granger causality (F–J) based on data recorded during anesthesia (row 1), sleep (row 2), immobile alertness (row 3), exploration (row 4), and rearing (row 5). Gray shaded regions indicate a non-Gaussian approximation of +/−1 standard deviation based on resampling (see methods). Bars above each peak indicates the range over frequency over which GC exceeds a 0.005 cutoff based upon permutation analysis; solid lines denote frequency regimes over which corticostriatal causality is above chance, and dashed lines denote regimes over which striatocortical causality is above chance. Note the dominance of cortical drive during both anesthesia and sleep, as well as the complete lack of causality peaks in the striatocortical direction during sleep. Also note the refined information revealed by Granger causality. The coherence spectra are similar among the three behaviors; however, the relative contribution of information flow in the corticostriatal and striatocortical directions varies substantially among behaviors.

We analyzed data from five freely behaving animals as well as data gathered under anesthesia in four of these five animals. Data were consistent across animals, and a large number of trials were collected for each behavior of interest to ensure statistical robustness. General results are shown in Table 1 and Fig. 2. Detailed analyses of each behavioral epoch are provided in Figs. 3–4. GC was derived by fitting a linear model to time-lagged values of each time series. To ensure that GC was robust against variations in model order, we inspected data sets generated using multiple model orders and found them to be very consistent. With the exception of anesthesia, the BIC converged between an order of 5 and 50 for all data sets. For data gathered during anesthesia, the BIC decreased monotonically over this range; however, GC computed using these data was consistent at model orders of 30, 40, and 50 hence we chose 40 as an appropriate trade-off between over-parameterization and sufficient spectral resolution. This method has been applied in past studies in which a parameterization criterion failed to converge [24]. Coherence between cortex and striatum varies across behavioral states (Fig. 4, A–D) and different patterns of directed influence as revealed by GC underlie coherence during different behaviors (Fig. 4, F–J).

### Power and Coherence Spectra Vary with Behavioral States

We characterized LFPs gathered during all behavioral states using standard spectral estimation techniques. The distribution of each signal's spectral content over frequency bins was quantified by estimating the power spectral density from the signal's autoregressive representation. Spectral interdependence between signals was determined by estimating the (magnitude squared) coherence, which is normalized and thus bound between zero (no interdependence) and one (complete synchronization). During both wakefulness and inactivity, coherence between M1 and dStr was between 0.5 and 0.9, suggesting strong synchronization between these structures in all the behavioral states studied in this experiment.

LFPs collected during anesthesia were characterized by high, narrow peaks on the power spectra in the 1–5 Hz range for both M1 and dStr (Fig. 3, A, B). The maximum power spectral density value was higher than that observed during any other behavioral state, and the total power was tightly concentrated in the 1–5 Hz range. The total amplitude of oscillatory activity in both structures is therefore relatively high during anesthesia and is accounted for by a very narrow range of low-frequency components. Oscillatory activity between these structures was strongly synchronized as indicated by the high coherence in the 1–5 Hz range with a smaller peak in the 10–20 Hz range (Fig. 4, A).

Natural sleep, like anesthesia, was characterized mainly by low-frequency oscillations in LFPs gathered from both M1 and dStr (Fig. 3, B, G). Also, as was the case during anesthesia, cortical and striatal LFPs exhibited strong coherence during sleep (Fig. 4, B). LFPs recorded during sleep did, however, differ from data gathered during anesthesia in both amplitude and spectral content (Fig. 3, F,G). The maximum power over all frequencies for LFPs gathered during sleep was about half that of anesthesia, indicating that the average magnitude of LFPs in these structures is smaller during natural sleep than anesthesia. Moreover, the roll-off in the power spectrum was gentler for data gathered during sleep, and the coherence spectrum decayed monotonically until converging at a minimum of about 0.4. In contrast, coherence spectra generated using data gathered under anesthesia exhibited a sharp drop-off at ∼5 Hz followed by a smaller, broader peak in the 10–20 Hz range (Fig. 4, A).

The maximum power spectral value, which quantifies the average amplitude of a signal at its dominant component frequency or frequencies, was largest for anesthesia and decreased by one half during natural sleep (Fig. 4). The maximum peak coherence value, conversely, was similar for LFPs gathered during both anesthesia and sleep (Fig. 4, A, B), suggesting that the average amplitude of LFPs in each structure, but not the degree of synchronization between them, differentiates anesthesia from sleep. LFPs recorded during wakefulness exhibited a substantially lower maximum power spectral value than those recorded during sleep and anesthesia. The maximum coherence was also reduced, although not as dramatically.

**Figure 5:**
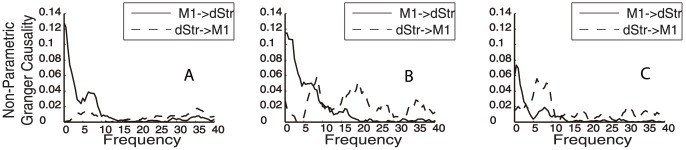
Non-parametric Granger causality during spontaneous behavior. Granger causality derived directly from the Fourier transform of LFPs gathered during alertness (A), exploration (B), and rearing (C). Note the substantial similarity between these estimates of Granger causality and those obtained using a regression model. The agreement between these measures lends credence to our analysis and rules out model fitting as a source of error.

LFPs gathered during behavior showed interesting similarities in their low frequency components across behavioral states (1–5 Hz), but exhibited different power levels in the 5–10 Hz and (in some cases) the ∼15 Hz range during different behaviors. During all four wakeful behaviors (alertness, exploration, rearing, and grooming) a substantial portion of the signal's power spectrum was accounted for by oscillations in the 1–5 Hz range (Fig. 3, C–E and H–J) and was accompanied by corresponding peaks in the coherence spectra (Fig. 4, C–E). As can be seen in Figs. 4 and 5, power and coherence in the 1–5 Hz range vary in their magnitude across difference behaviors; however, power and coherence in the 5–10 Hz range is the most marked differentiator of behavioral states.

### Identifying Directed Network Dynamics with Granger Causality

To address directed influence giving rise to corticostriatal coherence, which is the central issue of this paper, we applied GC to decompose the coherence spectra into two causal spectra, one characterizing the corticostriatal pathway and the other the striatocortical pathway. We differentiated two types of symmetry – or asymmetry – that can emerge in the GC spectra: spectral symmetry and directional symmetry. Spectral symmetry refers to how similar the GC spectra are in the distribution of their total power over different frequency bins. Directional symmetry refers to the relative contribution of each connection to the total causality spectrum.

Sleep and anesthesia were characterized by substantial directional asymmetry, and corticostriatal GC accounted for most of the coherence spectrum (Fig. 4, F, G). Though striatocortical GC is substantially less than corticostriatal GC during anesthesia, it is still above chance and accounts for a portion of both the 1–5 Hz and 10–15 Hz peaks in the coherence spectrum.

A coherence peak in the low frequency range was also observed during natural sleep, although this peak was broader with a gentler roll-off than the one characterizing anesthesia (Fig. 4, B). In addition, the corticostriatal GC spectrum closely tracked the coherence spectrum and was also broader and less sharp (Fig. 4, F, G). Interestingly, whereas data recorded during anesthesia exhibited distinct peaks in two frequency bands (Fig. 4, A, C), data recorded during natural sleep exhibited only a single peak followed by a monotonic decay in both the coherence and causality spectra (Fig. 4, B, G). Finally, in addition to the large corticostriatal causality peak observed during sleep, we also found that striatocortical causality in the 1 to 20 Hz range is above chance during sleep, though it is less than corticostriatal causality by an order of magnitude and does not exhibit substantial spectral peaks (Fig. 4, G).

Intriguingly, we found that for spontaneous behaviors the relative influence of striatum over cortex increases depending on the behavior. During alertness, when the rat is immobile but clearly awake and aware of its surroundings, cortical drive exceeds striatal drive, and accounts for both peaks in the coherence spectrum (Fig. 4, H). When the rat is actively exploring its surroundings, a behavior characterized by small movements and limited locomotion, striatal influence increases, particularly in the ∼5 Hz and ∼15 Hz range (Fig. 4, I). Finally, when the animals are rearing, a behavior that involves recruitment of more muscle groups and greater coordination than alertness or exploration, striatal drive in the ∼5 Hz and ∼15 Hz range actually exceeds cortical drive, though both remain above chance and thus contribute to the coherence spectrum (Fig. 4, J).

### Comparison to Nonparametric Techniques

In order to ensure that our data were robust against parameter estimation errors, we applied nonparametric GC (Dhamala et al., 2008) to the data. This method derives GC directly from the Fourier transform of the time series using the factorization of the spectral matrices (Wilson, 1972). We found excellent agreement between GC derived using parametric and nonparametric methods, lending credence to our results and suggesting that complications from model estimation did not significantly affect our results (Compare Fig. 4, H–J with Fig. 5).

### Spectral Stationarity During Anesthesia and Sleep

**Figure 6:**
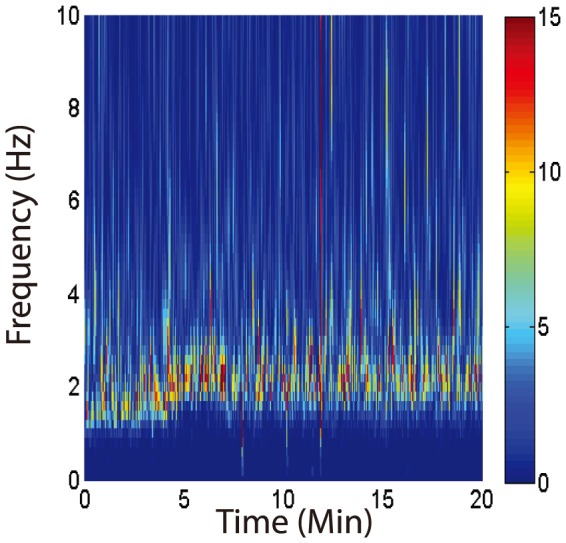
Time-frequency content of LFPs recorded in M1 during anesthesia in all rats showing consistent spectral content in multiple signals recorded in multiple animals. This figure was generated by wavelet transforming each epoch using multiwavelet estimation and estimating the average squared amplitude. The resulting single-trial spectrograms were concatenated to form the image shown. The color bar gives the averaged squared amplitude of the wavelet coefficients at each point in time-frequency space. Note the concentration of power in the 1–3 Hz range across all epochs of anesthesia.

Our decision to treat LFPs recorded during anesthesia as single long trials that could be split into epochs of arbitrary length rests on the assumption that the spectral content of the fields does not vary significantly across multiple stages of anesthesia and sleep. To validate this assumption, we constructed time-frequency plots of individual trials using a multiwavelet transform (Brittain et al., 2007) and concatenated consecutive trials into a single spectrogram. Inspecting these spectrograms, we were able to confirm that the spectral content of LFPs recorded during anesthesia and sleep is constant. The multiwavelet method produces ensemble averages from individual trials, and can thus produce unbiased estimates of power, by averaging over multiple pairwise independent estimates of the signal's squared amplitude in the space spanned by the generalized Morse wavelets (Olhede and Walden, 2002). A representative spectrogram, using data recorded in M1 during anesthesia, is shown in Fig. 6.

## Discussion

### Importance of main findings

Here, for the first time, we examined directed influence between M1 and dStr using animals engaged in voluntary behaviors. Contrary to our expectation, we found that effective connectivity became bidirectional in freely behaving animals. In agreement with previous reports, effective connectivity was unidirectional, from cortex to striatum, during natural sleep and anesthesia. Early theoretical perspectives, informed by the reentrant structure of the BG, emphasized the role of this system in refining and shaping cortical information for behavioral output [7], [13], while recent advances in BG modeling highlight the complex dynamic unfolding across the entire cortico-BG network [52]–[55]. Our results provide empirical support for models that emphasize dynamic interactions between cortex and subcortex as opposed to unidirectional drive. These results also highlight the refinements to conventional spectral analysis afforded by causality detection methods. Finally, we expect this approach will be useful for future work where it will be important to monitor effective connectivity under different movements or behavioral conditions.

### Interpretation and uses of effective connectivity

There is often a strong tendency for researchers to interpret the results of an effective connectivity measure in terms of physical connectivity. In the present study, it would seem natural to attribute strong GC in the corticostriatal pathway to the direct, monosynaptic, physical connections from cortex to striatum. While this may be the case, the experiments done here, by themselves, are not sufficient to justify this conclusion. Similarly, it is extremely likely that strong GC in the striatocortical pathway is produced by the known polysynaptic loop from striatum, through the BG, to thalamus and back to cortex. Again, this physical pathway may be contributing significantly to the measured GC, but the experiments reported here cannot establish this conclusion. The inability of GC to establish physical connectivity has been clearly documented in the elegant work of several researchers [56]–[58]. These papers demonstrate, for example, that common drive with different delays can produce strong GC measurements between recording sites even in the case where there are no direct synaptic connections. In spite of these constraints, effective connectivity metrics are powerful tools when their results are interpreted with appropriate caution, given the limited availability of data and the complexity of the systems under analysis. Our interpretations of our results are theoretically strong given current knowledge of the BG and cortex. That being said, further work is necessary to determine exactly how much of the GC observed between these nodes is mediated by their reciprocal connections and how much is accounted for by other sources of input.

### Granger causality differences during anesthesia and natural sleep

Although previous work has reported corticostriatal GC under anesthesia [20], our work extends these results by comparing GC under anesthesia to GC during natural sleep. The total amplitude of both cortical and striatal LFPs increased during inactivity relative to wakefulness along with a corresponding increase in their coherence. In tandem, these results show both that the magnitude of LFP oscillations and their synchrony across different structures increases during periods of natural and drug-induced inactivity. This is a well-established phenomenon, as low-frequency oscillations are known to characterize anesthesia and sleep in both cortex and striatum [59]. To date, no research has addressed the driver/receiver dynamics giving rise to these coherence patterns. We found that during both rest and anesthesia, GC in the corticostriatal direction exceeds the amplitude of the reciprocal connection by an order of magnitude. This result shows that, during inactivity, striatal oscillations are strongly driven by cortical input and that the increased low frequency power observed in striatal LFPs is largely independent of autonomous dynamics within striatum itself.

A possible explanation for this result is that, during rest, cortex drives a brain-wide network into a strongly synchronized resting state characterized by low frequency oscillations. The reciprocal connectivity from striatum suggests that subcortico-cortical feedback persists, even during anesthesia, but plays a substantially reduced role in network-level control. This result dovetails with prior research, which shows that during sleep and anesthesia thalamic neurons shift from transfer mode to burst mode due to hyperpolarization from reduced inputs [60]. This is known to attenuate the influence of sensory inputs to cortex via the thalamus. The increased causal power and sharpness of the GC peak associated with anesthesia relative to natural sleep suggests that strong, unidirectional coupling in a small range of frequencies is a key feature of anesthesia. We note that we only analyzed data gathered under ketamine/xylazine anesthesia. As different anesthetics have different mechanisms of action, it would be revealing to explore if and how different anesthetics differ in their effects on this systems. The observed difference between spontaneous and drug induced inactivity is interesting nevertheless.

### Granger causality differences during free behaviors

Here we have not only shown that, in keeping with previous studies, the frequency content of LFPs varies robustly with behavior, but we have also demonstrated for the first time that reciprocal influence of striatum over cortex is a key feature of information processing during voluntary behavior.

Spontaneous behaviors differ substantially in their causality spectra as well as their coherence spectra. Though the total power remained relatively constant (∼1.5–2.0 a.u.) across all behaviors, the relative contributions of low and high frequency components varied robustly across behaviors. Coherence in the 1–5 Hz range persisted across all trials, but the magnitude of the 5–10 Hz and 15–20 Hz components increased during exploration and rearing. Though further work is required to fully elucidate the physiological significance of LFPs, our findings bolster the prevailing view that they are robust neurological markers of behavioral events and thus provide important information about neuronal processes [15], [61], [62]. Importantly, we found that corticostriatal and striatocortical influence contribute to different components of the coherence spectra. For instance, low frequency power in striatum arises largely from cortical input across all behavioral states, whereas coherence in higher frequency bands arises from both corticostriatal and striatocortical interactions. In fact, striatal influence over cortex in the 5–10 Hz band exceeds that of cortex over striatum, though the GC in both directions remains above chance.

The total interdependence between cortex and striatum, as measured by the sum of GC values across all frequencies, is relatively stable across behaviors; however, the relative magnitude of the low frequency components decreases, while concordantly the contribution of higher frequency components to the overall coherence and causality spectra increases. The shift of power and causal influence from low to high frequency bands during periods of motor output is particularly interesting when considered in tandem with our findings during sleep and anesthesia. Slow wave activity is a well-known marker of anesthesia and general inactivity. Moreover, our group has found evidence that the emergence of hyperkinesia in HD mice is associated with increased power in the ∼25 Hz range [8]. Fast and slow wave oscillations are associated with a variety of behaviors and our data suggest that they play different roles in the facilitation of information flow between two motor processing regions. Due to the diversity of physiological phenomena that contribute to LFPs, it is difficult to know the exact relevance of different frequency bands at the neuronal level. Nevertheless, the concordance between LFPs and behavioral output is striking, and recent work has expanded our understanding of how LFPs can facilitate neuronal processing [63].

We emphasize that our results are all based on data gathered from freely-behaving, untrained animals. Knowing the neural correlates of untrained behaviors is critical to understanding the role of the BG in selecting among multiple possible courses of action. We have shown that distinct coherence patterns characterize different spontaneous behaviors. Moreover, we have applied novel methodologies to elucidate the causal dynamics giving rise to coherence between cortex and striatum. Combining coherence and effective connectivity in the analysis of data gathered during free behavior allowed us to track changes in information flow as animals spontaneously transition among different behaviors.

### Equal contributions of cortex and striatum during free behavior

Our data clearly indicate that behaviorally relevant coherence between cortex and striatum is mediated equally by effective connections in both directions. These systems share extensive synaptic connections that surely account for a portion of the information flow observed in this study. At the same time, other sources of input, such as time-lagged input from thalamus, may artificially inflate the observed causality and further experiments are required to address this issue. Though we cannot address sources of striatocortical causality using these data, further lines of research can clarify this issue. For example recent findings by Saalmann and colleagues show that a thalamic system exerts substantial behaviorally-relevant influence over intra-cortical processing during a selective attention task [44]. As striatum influences cortex via thalamic output neurons, Saalmann et al.'s results support our findings and suggest a mechanism of reciprocal information flow from striatum to cortex.

### Conclusions

We have presented an assessment of interactions between M1 and dStr during spontaneous behaviors and various stages of inactivity. Using GC, we provide new insight into the network dynamics underlying coherence between these structures. We found that anesthesia and rest are accompanied by increased corticostriatal drive as opposed to decoupling, suggesting an important role of cortico-subcortical input in maintaining resting state dynamics. Furthermore, we provide insight into how interactions between M1 and dStr produce voluntary, non-evoked behaviors. We caution that while LFPs are robust markers of behavior output, they have a complex and not fully understood relationship to spiking activity from individual neurons that warrants further attention. Nevertheless, LFPs have great value as they reflect large-scale activity across hundreds of neurons, making them well suited for the study of population-level dynamics. We provide evidence that GC is a valuable tool in the study of neural networks in keeping with a rapidly growing body of literature. Lastly, our results and design can be built upon to extend the scope of research into the BG and other systems.
